# Experiences of Unstable Housing Among High School Students — Youth Risk Behavior Survey, United States, 2021

**DOI:** 10.15585/mmwr.su7201a4

**Published:** 2023-04-28

**Authors:** Izraelle I. McKinnon, Kathleen H. Krause, Leah Robin, Adriane King, Michelle Leon-Nguyen, Evelyn Zavala, Nicolas A. Suarez, Connie Lim, Jennifer Smith-Grant, J. Michael Underwood

**Affiliations:** ^1^Division of Adolescent and School Health, National Center for HIV, Viral Hepatitis, STD, and TB Prevention, CDC; ^2^Epidemic Intelligence Service, CDC; ^3^U.S. Public Health Service

## Abstract

Youths experiencing unstable housing face higher risks for poor physical, mental, and sexual health outcomes and increased risk for suicide compared with their peers experiencing stable housing. In addition, youths of color and sexual minority youths are disproportionately more likely to experience homelessness. For the first time, in 2021, the nationally representative Youth Risk Behavior Survey included an item assessing housing stability, or nighttime residence among students in grades 9–12 in the United States. During 2021, 2.7% of U.S. high school students experienced unstable housing. Among racial and ethnic subgroups, Native Hawaiian or other Pacific Islander youths were most likely to experience unstable housing, followed by American Indian or Alaska Native and Black youths. Sexual minority (lesbian, gay, bisexual, and questioning or other) youths were more likely to experience unstable housing compared with their heterosexual peers. Compared with students who were stably housed, students who were unstably housed were more likely to engage in risky sexual behaviors, substance use, and suicide ideation and attempts, and to experience violence. These findings highlight which adverse health risks and behaviors are elevated among youths experiencing housing insecurity. Focused public health interventions are required to address the disproportionate burden of health risks prevalent among youths who are unstably housed.

## Introduction

According to the National Center for Homeless Education, during the 2020–2021 school year, approximately 1.1 million youths in prekindergarten through 12th grade, or 2.2% of all students, experienced unstable housing (i.e., lacked a fixed, regular, and adequate nighttime residence) in the United States (*1*). Studies have documented the burden of adverse health risks and behaviors among youths who are unstably housed, including more high-risk sexual behaviors and experiences of violence (including partner abuse), when compared with youths who are stably housed (*2*). Youths experiencing unstable housing report higher levels of psychiatric disorders, suicide ideation and attempts, and substance use when compared with their peers (*2*,*3*). Among students experiencing unstable housing, students of color are overrepresented, with the exception of Asian students (https://nche.ed.gov/wp-content/uploads/2021/12/Student-Homelessness-in-America-2021.pdf). In addition, because sexual minority youths (lesbian, gay, bisexual, and questioning or other [LGBQ+]) might face family rejection and mistreatment because of their sexual identity, studies often document higher rates of housing instability among this population compared with heterosexual youths (https://www.thetrevorproject.org/wp-content/uploads/2022/02/Trevor-Project-Homelessness-Report.pdf). Disparities in housing stability place students of color and sexual minority youths at higher risk for behaviors and health outcomes associated with experiences of unstable housing.

A 2019 report using state and local Youth Risk Behavior Survey (YRBS) data demonstrated a higher prevalence of risk behaviors among youths who were unstably housed (*4*). Students experiencing unstable housing were more likely to be male, non-Hispanic Black, and identify as lesbian, gay, or bisexual. Students who were unstably housed were more likely to report substance use, be currently sexually active, not use a condom during last sexual intercourse, experience violence victimization, and report suicide ideation and attempts compared with students who were stably housed (*4*). Although that study illustrates a disproportionate burden of adverse health risk behaviors and outcomes among youths experiencing unstable housing compared with youths with stable housing, the data are representative of only 23 states and 11 local school districts. This report provides 2021 YRBS results on housing instability among high school students, the first report using nationally representative data. Public health professionals, advocates, and policy makers can use these data to assess demographic characteristics, risk behaviors, and public health needs among youths experiencing unstable housing. These findings can be used to guide evidence-based interventions for the vulnerable populations of youths that experience unstable housing.

## Methods

### Data Source

This report includes data from the 2021 YRBS (N = 17,232), a cross-sectional, school-based survey conducted biennially since 1991. Each survey year, CDC collects data from a nationally representative sample of public and private school students in grades 9–12 in the 50 U.S. states and the District of Columbia. Additional information about YRBS sampling, data collection, response rates, and processing is available in the overview report of this supplement (*5*). The prevalence estimates for students experiencing unstable housing for the overall study population and by sex, race and ethnicity, grade, and sexual identity are available at https://nccd.cdc.gov/youthonline/App/Default.aspx. The full YRBS questionnaire is available at https://www.cdc.gov/healthyyouth/data/yrbs/pdf/2021/2021-YRBS-National-HS-Questionnaire.pdf. This activity was reviewed by CDC and was conducted consistent with applicable federal law and CDC policy.*

### Measures

To obtain information on housing stability, students were asked, “During the past 30 days, where did you usually sleep?” Responses were coded into a binary variable of experiencing unstable housing (“in the home of a friend, family member, or other person because I had to leave my home or my parent or guardian cannot afford housing,” “in a shelter or emergency housing,” “in a motel or hotel,” “in a car, park, campground, or other public place,” or “I do not have a usual place to sleep”) versus stable housing (“in my parent's or guardian's home,” or “somewhere else”). The response option “somewhere else” is included in the definition of “experiencing stable housing” because it provides students a response option to the question if they do not fully understand the question or response options, or do not fit squarely into any of the response option categories (e.g., students living in dormitories and students with recently deceased or incarcerated parents living with a relative) or within the definition of experiencing unstable housing. Student demographic characteristics included sex (female or male), grade (9, 10, 11, or 12), race (American Indian or Alaska Native [AI/AN], Asian, Black, Native Hawaiian or other Pacific Islander [NH/OPI], Hispanic, White, and persons of multiple races [multiracial]), and sexual identity (heterosexual or LGBQ+). (Persons of Hispanic origin might be of any race but are categorized as Hispanic; all racial groups are non-Hispanic.) Health risk behaviors used in this report, including those related to substance use, sexual health, violence victimization, and mental health and suicide risk, are provided (Table 1).

**TABLE 1:** Measures for selected health risk behaviors among high school students — Youth Risk Behavior Survey, United States, 2021

Variable	Question	Response options	Analytic coding
**Substance Use**			
Ever misused prescription opioids	During the past 30 days, how many times did you take prescription pain medicine without a doctor's prescription or differently than how a doctor told you to use it?	0 times, 1 or 2 times, 3–9 times, 10–19 times, 20–39 times, ≥40 times	Yes (1 or 2 times, 3–9 times, 10–19 times, 20–39 times, ≥40 times) versus no (0 times)
Ever used illicit drugs	During your life, how many times have you A) used any form of cocaine, including powder, crack, or freebase? B) sniffled glue, breathed the contents of aerosol spray cans, or inhaled any paints or sprays to get high? C) used heroin (also called smack, junk, or China White)? D) used methamphetamines (also called speed, crystal meth, crank, ice, or meth)? E) used ecstasy (also called MDMA or Molly)? F) used hallucinogenic drugs, such as LSD, acid, PCP, angel dust, mescaline, or mushrooms?	0 times, 1 or 2 times, 3–9 times, 10–19 times, 20–39 times, ≥40 times	Yes (1 or 2 times, 3–9 times, 10–19 times, 20–39 times, ≥40 times [for any included drug]) versus no (0 times [for all included drugs])
Ever injected any illegal drug	During your life, how many times have you used a needle to inject any illegal drug into your body?	0 times, 1 time, ≥2 times	Yes (1 time, ≥2 times) versus no (0 times)
**Sexual health**			
Currently sexually active	During the past 3 months, with how many people did you have sexual intercourse?	I have not had sexual intercourse, I have had sexual intercourse, but not during the past 3 months, 1 person, 2 persons, 3 persons, 4 persons, 5 persons, ≥6 persons	Yes (1 person, 2 persons, 3 persons, 4 persons, 5 persons, ≥6 persons) versus no (I have not had sexual intercourse, I have had sexual intercourse, but not during the past 3 months)
Used alcohol or drugs at last sexual intercourse	Did you drink alcohol or use drugs before you had sexual intercourse the last time?	I have never had sexual intercourse, yes, no	Yes versus no (no, I have never had sexual intercourse)
Did not use a condom during last sexual intercourse	The last time you had sexual intercourse, did you or your partner use a condom?	I have never had sexual intercourse, yes, no	Yes versus no (no, I have never had sexual intercourse)
Not tested for any STD (other than HIV) in the past 12 months	During the past 12 months, have you been tested for a sexually transmitted disease (STD) other than HIV, such as chlamydia or gonorrhea?	Yes, no, not sure	No (no, not sure) versus yes
Not ever tested for HIV	Have you ever been tested for HIV, the virus that causes AIDS? (Do not count tests done if you donated blood.)	Yes, no, not sure	No (no, not sure) versus yes
**Violence victimization**			
Experienced sexual dating violence in the past 12 months	During the past 12 months, how many times did someone you were dating or going out with force you to do sexual things that you did not want to do? (Count such things as kissing, touching, or being physically forced to have sexual intercourse.)	I did not date or go out with anyone during the past 12 months, 0 times, 1 time, 2–3 times, 4–5 times, ≥6 times	Yes (1 time, 2–3 times, 4–5 times, ≥6 times) versus no (0 times, I did not date or go out with anyone during the past 12 months)
Experienced physical dating violence in the past 12 months	During the past 12 months, how many times did someone you were dating or going out with physically hurt you on purpose? (Count such things as being hit, slammed into something, or injured with an object or weapon.)	I did not date or go out with anyone during the past 12 months, 0 times, 1 time, 2–3 times, 4–5 times, ≥6 times	Yes (1 time, 2–3 times, 4–5 times, ≥6 times) versus no (0 times, I did not date or go out with anyone during the past 12 months)
Experienced sexual violence by anyone in the past 12 months	During the past 12 months, how many times did anyone force you to do sexual things that you did not want to do? (Count such things as kissing, touching, or being physically forced to have sexual intercourse.)	0 times, 1 time, 2–3 times, 4–5 times, ≥6 times	Yes (1 time, 2–3 times, 4–5 times, ≥6 times) versus no (0 times)
**Mental health and suicide risk**			
Poor mental health in the past 30 days	During the past 30 days, how often was your mental health not good? (Poor mental health includes stress, anxiety, and depression.)	Never, rarely, sometimes, most of the time, always	Yes (rarely; sometimes; most of the time; always) versus no (never)
Experienced persistent feelings of sadness or hopelessness	During the past 12 months, did you ever feel so sad or hopeless almost every day for two weeks or more in a row that you stopped doing some usual activities?	Yes, no	Yes versus no
Seriously considered suicide in the past 12 months	During the past 12 months, did you ever seriously consider attempting suicide?	Yes, no	Yes versus no
Made a suicide plan in the past 12 months	During the past 12 months, did you make a plan about how you would attempt suicide?	Yes, no	Yes versus no
Attempted suicide	During the past 12 months, how many times did you actually attempt suicide?	0 times, 1 time, 2–3 times, 4–5 times, ≥6 times	Yes (1 time, 2–3 times, 4–5 times, ≥6 times) versus no (0 times)

### Analysis

Prevalence estimates were calculated for all responses regarding where a student usually slept in the past 30 days; the dichotomized behavior of being unstably versus stably housed; and for experiencing unstable housing versus being stably housed stratified by sex, grade, race and ethnicity, and sexual identity. Logistic regression models with specifications for predicted marginal proportions provided unadjusted and adjusted (for demographic characteristics not being examined in that model [i.e., sex, grade, race and ethnicity, and sexual identity]) prevalence ratios to detect disparities within a demographic category compared with a common reference group (e.g., all grades compared with grade 9). Prevalence estimates of each risk behavior were stratified by housing status. Unadjusted and adjusted (for all demographic characteristics) prevalence ratios were calculated to compare the risk for a health risk behavior among students who were unstably housed with those who were stably housed. Statistical significance was determined if the 95% CI did not cross the null value of 1.0. Analyses were conducted using SUDAAN (version 11.0.3; RTI International) to account for complex survey design and nonresponse.

## Results

In 2021, 2.7% of high school students in the United States were unstably housed in the 30 days before participating in YRBS: 1.7% of high school students slept in the home of a friend, family member, or other person because they had to leave their home or their parent or guardian could not afford housing; 0.3% slept in a shelter or emergency housing; 0.2% slept in a motel or hotel; 0.2% slept in a car, park, campground, or other public place; and 0.3% did not have a usual place to sleep. Approximately 97% of high school students were stably housed, with 96.1% of students sleeping in their parent’s or guardian’s home and 1.3% sleeping somewhere else (Table 2).

**TABLE 2:** Prevalence of unstable and stable housing among high school students —Youth Risk Behavior Survey, United States, 2021

Nighttime residence	% (95% CI)*
** Housing options^†^**	
In the home of a friend, family member, or other person because I had to leave my home or my parent or guardian cannot afford housing	1.7 (1.3–2.1)
In a shelter or emergency housing	0.3 (0.2–0.4)
In a motel or hotel	0.2 (0.1–0.3)
In a car, park, campground, or other public space	0.2 (0.1–0.4)
I do not have a usual place to sleep	0.3 (0.2–0.4)
In my parent's or guardian's home	96.1 (95.2–96.7)
Somewhere else	1.3 (0.7–2.3)
**Housing stability status^§^**	
Unstable housing	2.7 (2.2–3.3)
Stable housing	97.3 (96.8–97.8)

* N = 17,232 respondents. Because the state and local questionnaires differ by jurisdiction, students in these schools were not asked all national YRBS questions. Therefore, the total number (N) of students answering each question varied. Percentages in each category are calculated on the known data.

^†^ Student response to, “During the past 30 days, where did you usually sleep?”

^§^ Housing items were dichotomized as unstable housing for responses “in the home of a friend, family member, or other person because I had to leave my home or my parent or guardian cannot afford housing," "in a shelter or emergency housing," "in a motel or hotel," "in a car, park, campground, or other public space," or "I do not have a usual space to sleep" and stable housing for responses "in my parent’s or guardian’s home" or "somewhere else."

Female and male students were similarly likely to have experienced unstable housing in the past 30 days (2.4% of female and 2.7% of male students) (Table 3). The prevalence of unstable housing increased with increasing grade level; 2.0% of 9th graders, 2.1% of 10th graders, 2.8% of 11th graders, and 3.4% of 12th graders were unstably housed. Adjusted for other demographic variables, the prevalence of unstable housing was 1.7 times higher among 12th-grade compared with 9th-grade students. The racial and ethnic groups that experienced the highest prevalence of unstable housing were NH/OPI (10.0%), AI/AN (7.9%), and Black (5.1%) students; after adjusting for other demographic variables, these groups experienced 5.9, 4.7, and 2.6 times higher prevalence of unstable housing compared with White students, respectively. The prevalence of unstable housing was lowest among Asian students (0.8%); after adjusting for other demographic variables, Asian students experienced 0.3 times lower prevalence of unstable housing compared with White students. Among sexual identity groups, the prevalence of unstable housing was 2.0% among heterosexual students, 4.7% among lesbian or gay students, 4.2% among bisexual students, 4.0% among questioning students, and 2.6% among students who describe their sexual identity in some other way. Overall, the prevalence of unstable housing was approximately two times higher among students who identify as LGBQ+ compared with heterosexual students.

**TABLE 3:** Prevalence of unstable housing among high school students, by select student characteristics — Youth Risk Behavior Survey, United States, 2021

Characteristic	% (95% CI)*	PR (95% CI)	aPR (95% CI)^†^
**Sex**			
Female	2.4 (1.9–3.2)	Ref	Ref
Male	2.7 (2.2–3.4)	1.1 (0.8–1.5)	1.2 (0.8–1.7)
**Grade**			
9	2.0 (1.4–2.9)	Ref	Ref
10	2.1 (1.5–2.8)	1.0 (0.7–1.5)	0.9 (0.6–1.5)
11	2.8 (2.0–4.0)	1.4 (0.9–2.3)	1.4 (0.9–2.2)
12	3.4 (2.7–4.3)	1.7 (1.1–2.8)^§^	1.7 (1.1–2.8)^§^
**Race and ethnicity** ^¶^			
American Indian or Alaska Native	7.9 (4.1–14.4)	3.9 (1.9–7.7)^§^	4.7 (2.3–9.8)^§^
Asian	0.8 (0.4–1.5)	0.4 (0.2–0.8)^§^	0.3 (0.1–0.8)^§^
Black	5.1 (3.8–6.8)	2.5 (1.8–3.4)^§^	2.6 (1.9–3.7)^§^
Native Hawaiian or other Pacific Islander	10.0 (4.3–21.7)	4.9 (2.1–11.7)^§^	5.9 (2.6–13.2)^§^
White	2.0 (1.6–2.5)	Ref	Ref
Hispanic	2.5 (1.6–3.9)	1.2 (0.7–2.2)	1.1 (0.7–1.9)
Multiracial	3.1 (2.0–5.0)	1.5 (0.9–2.6)	1.5 (0.9–2.8)
**Sexual identity**			
Heterosexual	2.0 (1.6–2.5)	Ref	Ref
Gay or lesbian	4.7 (2.9–7.3)	2.4 (1.5–3.7)^§^	1.9 (1.0–3.3)^§^
Bisexual	4.2 (2.6–6.6)	2.1 (1.4–3.3)^§^	2.3 (1.4–3.7)^§^
Questioning	4.0 (2.2–7.1)	2.0 (1.2–3.5)^§^	1.8 (1.1–3.1)^§^
Other sexual identity	2.6 (1.6–4.2)	1.3 (0.8–2.2)	1.5 (0.9–2.6)

**Abbreviations:** aPR = adjusted prevalence ratio; PR = prevalence ratio; Ref = referent group.

* N = 17,232 respondents. Because the state and local questionnaires differ by jurisdiction, students in these schools were not asked all national YRBS questions. Therefore, the total number (N) of students answering each question varied. Percentages in each category are calculated on the known data.

^†^ PR for sex adjusted for grade, race/ethnicity, and sexual identity; PR for grade adjusted for sex, race and ethnicity, and sexual identity; PR for race/ethnicity adjusted for sex, grade, and sexual identity; PR for sexual identity adjusted for sex, grade, and race and ethnicity.

^§^ A significant difference in the prevalence of unstable housing across levels of the demographic characteristics was observed. Differences were considered significant if the 95% CI did not cross the null value of 1.0.

^¶^ Persons of Hispanic or Latino (Hispanic) origin might be of any race but are categorized as Hispanic; all racial groups are non-Hispanic.

Students who were unstably versus stably housed were more likely to have ever misused prescription opioids (36.4% versus 11.7%, respectively), used illicit drugs (41.7% versus 12.7%, respectively), and injected any illegal drug (22.5% versus 0.9%, respectively) (Table 4). After adjusting for demographics, the prevalence of prescription opioid misuse and illicit drug use was nearly three times higher among students who were unstably versus stably housed and 19 times higher for injection drug use. The prevalence of current sexual activity and use of alcohol or drugs at last sexual intercourse was two times higher among students who were unstably versus stably housed. The prevalence of not using a condom at last sexual intercourse was higher among students who were unstably housed; however, after adjusting for demographic variables, the prevalence ratio comparing students who were unstably with stably housed was no longer significant. The prevalence of students who did not get tested for sexually transmitted diseases (STDs) in the past year or who had never been tested for HIV among students experiencing unstable housing was lower (81.2% and 81.4%, respectively) compared with those experiencing stable housing (95.2% and 94.7%, respectively). There were also differences in the prevalence of sexual and physical dating violence and sexual violence by anyone in the past year by housing stability status; 28.5% of students who were unstably housed experienced sexual dating violence, 31.9% experienced physical dating violence, and 27.6% experienced sexual violence by anyone, compared with 9.3%, 7.7%, and 10.6% among those who experienced stable housing, respectively. The prevalence of these forms of violence victimization was two to nearly four times higher among students who were unstably versus stably housed.

**TABLE 4:** Prevalence of select health risk behaviors, by housing stability status among high school students — Youth Risk Behavior Survey, United States, 2021

Behavior	Unstable % (95% CI)*	Stable % (95% CI)*	PR (95% CI)^†^	aPR (95% CI)^†,§^
**Substance use**				
Ever misused prescription opioids	36.4 (30.3–43.0)	11.7 (10.8–12.6)	3.1 (2.6–3.7)^¶^	2.9 (2.4–3.4)^¶^
Ever used illicit drugs	41.7 (35.9–47.7)	12.7 (11.5–13.9)	3.3 (2.7–4.0)^¶^	3.0 (2.4–3.7)^¶^
Ever injected any illegal drug	22.5 (16.6–29.7)	0.9 (0.6–1.3)	25.9 (14.7–45.8)^¶^	19.0 (9.9–36.5)^¶^
**Sexual health**				
Currently sexually active	48.2 (41.7–54.8)	20.0 (18.7–21.3)	2.4 (2.1–2.8)^¶^	2.0 (1.6–2.6)^¶^
Used alcohol or drugs at last sexual intercourse	41.0 (28.2–55.2)	19.7 (17.5–22.2)	2.1 (1.5–3.0)^¶^	2.1 (1.5–3.1)^¶^
Did not use a condom during last sexual intercourse	60.2 (49.8–69.7)	47.6 (44.9–50.3)	1.3 (1.1–1.5)^¶^	1.2 (1.0–1.4)
Not tested for any STD (other than HIV) in the past 12 months	81.2 (76.7–85.0)	95.2 (94.5–95.9)	0.9 (0.8–0.9)^¶^	0.9 (0.8–1.0)^¶^
Not ever tested for HIV	81.4 (78.1–84.3)	94.7 (94.0–95.3)	0.9 (0.8–0.9)^¶^	0.9 (0.9–0.9)^¶^
**Violence victimization**				
Experienced sexual dating violence in the past 12 months	28.5 (18.6–41.0)	9.3 (8.2–10.5)	3.1 (2.2–4.3)^¶^	3.0 (2.1–4.5)^¶^
Experienced physical dating violence in the past 12 months	31.9 (25.6–39.0)	7.7 (6.6–8.8)	4.2 (3.2–5.4)^¶^	3.7 (2.9–4.8)^¶^
Experienced sexual violence by anyone in the past 12 months	27.6 (23.0–32.6)	10.6 (9.6–11.6)	2.6 (2.1–3.2)^¶^	2.6 (2.0–3.4)^¶^
**Mental health and suicide**				
Poor mental health in the past 30 days	35.5 (29.3–42.3)	29.0 (27.4–30.7)	1.2 (1.0–1.5)	1.1 (0.8–1.5)
Experienced persistent feelings of sadness or hopelessness in the past 12 months	56.8 (50.4–63.0)	42.6 (41.3–44.0)	1.3 (1.2–1.5)^¶^	1.3 (1.1–1.5)^¶^
Seriously considered suicide in the past 12 months	44.9 (40.1–49.9)	21.8 (20.7–22.9)	2.1 (1.8–2.4)^¶^	1.9 (1.6–2.3)^¶^
Made a suicide plan in the past 12 months	38.0 (30.9–45.6)	17.3 (16.0–18.7)	2.2 (1.7–2.8)^¶^	2.0 (1.5–2.6)^¶^
Attempted suicide in the past 12 months	38.2 (31.7–45.2)	9.4 (8.6–10.2)	4.1 (3.3–5.0)^¶^	3.3 (2.5–4.2)^¶^

**Abbreviations:** aPR = adjusted prevalence ratio; PR = prevalence ratio; STD = sexually transmitted disease.

* N = 17,232 respondents. Because the state and local questionnaires differ by jurisdiction, students in these schools were not asked all national YRBS questions. Therefore, the total number (N) of students answering each question varied. Percentages in each category are calculated on the known data.

^†^ Comparing the health behavior prevalence among students experiencing unstable housing versus stable housing.

^§^ Adjusted for sex, grade, race and ethnicity, and sexual identity.

^¶^ A significant difference in prevalence of a health behavior by housing stability status was observed. Differences were considered significant if the 95% CI did not cross the null value of 1.0.

The prevalence of poor mental health in the past 30 days was approximately the same among students who were unstably (35.5%) and stably (29.0%) housed in the past 30 days, although the prevalence of persistent feelings of sadness or hopelessness in the past year was significantly higher among students who experienced unstable housing (56.8%) compared with their peers who were stably housed (42.6%) (Table 4). Adjusting for other demographic variables, students who experienced unstable housing were nearly twice as likely to have seriously considered suicide or made a suicide plan during the past year, and more than three times as likely to have attempted suicide during the past year.

## Discussion

This report provides the first nationally representative estimates of housing instability among high school students and provides evidence that they were at higher risk for a broad range of health risk behaviors and adverse experiences compared with their peers who were stably housed. During 2021, 2.7% of all high school students experienced unstable housing in the 30 days before participating in YRBS. Furthermore, when compared with their peers who experienced stable housing, these students were more likely to engage in substance use, risky sexual health behaviors, and suicide ideation and attempts, and to experience violence. Youths experiencing unstable housing had a disproportionate burden of adverse health risks and behaviors that should be addressed with interventions that prevent experiencing unstable housing or ameliorate the adverse consequences of experiencing unstable housing.

The 2.7% prevalence of youths experiencing unstable housing estimated using YRBS data is consistent with other studies. A national estimate of all school-aged youths found that 2.2% of students were homeless (*1*), and a national study of households with youths aged 13–17 years found that 3.0% reported experiences of unstable housing (*6*). These estimates might differ slightly because of differences in groups of youths across studies, methods of identification, and data collection methods. YRBS specifically asks about unstable housing in the past 30 days and therefore might underestimate the prevalence of unstable housing among high school students over the course of the entire year of 2021. In addition, as evidenced in this report, NH/PI, AI/AN, Black, and LGBQ+ students were more likely to experience unstable housing than their white and heterosexual peers, and the prevalence of unstable housing increased with increasing grade level. The overrepresentation of racial and ethnic and sexual minority groups among youths experiencing housing instability also was consistent with other studies (*6*–*8*). However, unlike other studies, this study did not find an elevated risk for experiencing unstable housing among Hispanic students relative to their White peers (*6*). Furthermore, the higher prevalence of certain risk behaviors and experiences among youths who were unstably versus stably housed documented in the 2021 YRBS data were consistent with previous reports (*2*–*4*,*9*). Although youths who were unstably housed were more likely to be currently sexually active and use alcohol or other substances before last sex, they were also more likely to have ever been tested for HIV or any other STD in the past year. Among youths experiencing unstable housing, the prevalence of testing was higher among those who were currently sexually active and experiencing physical or sexual dating violence (*10*), risk behaviors which were prevalent among one fourth to nearly one half of the youths who were unstably housed in this sample.

Underlying certain experiences of youth homelessness are experiences of family instability, engagement in foster care, and situations of abuse and neglect. Youths who are unstably housed might have marginal support systems and be more likely to experience adverse childhood events (*11*), family rejection, and family instability (*12*) than their peers who are stably housed. Such adverse experiences and behaviors are associated with poorer health status among youths who are unstably versus stably housed (*11*). Intersecting identities might heighten these risks; for example, lesbian, gay, and bisexual homeless youths experience significantly more sexual victimization, depression, and anxiety than their heterosexual peers experiencing homelessness (*13*). Some experiences of homelessness among youths might be facilitated by service gaps or referrals to services that require relocation to access them, particularly in rural communities (*7*). Such residential instability is associated with a heightened likelihood of violence victimization (*12*). Despite increased exposure to adverse events and associated adverse physical, mental, and sexual health outcomes, episodes of unstable housing might serve as barriers to accessing consistent and comprehensive health and mental health care. Because of the intersection of housing instability trauma, lack of support, and fragmented and complicated access to health and mental health care, further research is needed to identify programs and policies to meet the health and mental health needs and complex life circumstances of youths experiencing homelessness.

Schools play a pivotal role in providing care and services to youths who are unstably housed. The McKinney-Vento Homeless Assistance Act (MVA) authorizes direct services that enable youths experiencing unstable housing to enroll, attend, and achieve success in school (http://uscode.house.gov/view.xhtml?path=/prelim@title42/chapter119/subchapter6/partB&edition=prelim). MVA-appointed liaisons serve as one of the primary contacts between families experiencing unstable housing and school support services and ensure that youths who are unstably housed can succeed academically. Certain MVA programs provide training and support that foster connections with families and youths through various comprehensive services for youths with complex needs, including referrals to school- and community-based programs for family counseling, adolescent health care, mental health, and LGBQ+ programs supported by student-led groups like Gender and Sexuality Alliances (GSAs). For example, Boston Public Schools established the Homeless Education Resource Network to refer students and families to housing service organizations (*14*). In New York City Public Schools, an annual MVA training is provided to 1,600 liaisons supporting students in temporary housing. Statewide efforts in Florida demonstrate how MVA funding is allocated to local education agencies (*15*), including Hillsboro County’s Help Students in Transition Program and the Broward County Homeless Education Assistance Resource Team.

## Limitations

General limitations for the YRBS are available in the overview report of this supplement (*5*). The findings in this report are subject to at least four additional limitations. First, causality cannot be inferred between housing instability and health risk behaviors because data are cross-sectional. Second, all variables did not share a common time reference point. Exposure to unstable housing was measured during the past 30 days, but the risk behaviors in this report included behaviors in which a student had ever engaged, behaviors that occurred during the past 12 months, and behaviors that occurred at time of last sexual intercourse. Third, this analysis is limited to students who attend school and were present on the day(s) of data collection; adolescents experiencing unstable housing are more likely to disengage from school (*16*). Finally, students who slept “somewhere else” in the past 30 days were categorized as experiencing stable housing, although students who experienced unstable housing might have also selected this option. Therefore, the prevalence of unstable housing might be underestimated, and prevalence ratios are conservative and reflect robust associations.

## Future Directions

School-based programmatic supports are important, and YRBS data can be used to inform these programs and resources and to enhance connections between MVA programs and other school-based resources, such as school-based health centers, mental health services, and GSAs. Resources for supporting youths who experience housing instability also should include guidance for reducing health risks and increasing access to clinical and mental health support. Policy efforts also should be made to prevent unstable housing among youths and address health risks among this population. For example, Chicago Public School’s 2022 Comprehensive Mental Health and Suicide Prevention Policy requires that a behavioral health team be established in every K–12 school to reduce stigma and increase staff ability to recognize students who are at risk for suicidality and mental health issues such as depression, noting housing instability as a root cause of such health risks (*17*). Additional research is needed to further explore the determinants that place unstably housed youths at risk for adverse health behaviors and experiences, as well as factors that might promote resilience among youths experiencing unstable housing. Schools could also consider interventions focusing on AI/AN, Black, NH/PI, and LGBQ+ students, who face a disproportionate burden of unstable housing.

## Conclusion

Students who are unstably housed are more likely to engage in health risk behaviors and encounter adverse experiences and are at greater risk for adverse outcomes when compared with their peers who are stably housed. This analysis underscores an ongoing need to assess the prevalence, characteristics, health risks, and adverse experiences of students experiencing unstable housing. To that end, YRBS will continue to collect data on youths experiences of unstable housing and track trends over time through national, state, and local surveys. Furthermore, some students might be more likely to experience unstable housing based on characteristics that reflect systems of power, such as race and ethnicity or sexual identity. MVA provides an example of guidance and programs to meet short- and long-term needs of unstably housed students and their families. Furthermore, school staff might consider implementing policies and practices for housing, insurance, legal needs, and health and mental health services for students experiencing unstable housing.

## Footnotes

* See e.g., 45 C.F.R. part 46.102(l)(2), 21 C.F.R. part 56; 42 U.S.C. §241(d); 5 U.S.C. §552a; 44 U.S.C. §3501 et seq.
